# Dutch Translation and Adaptation of the Treatment Beliefs Questionnaire for Chronic Pain Rehabilitation

**DOI:** 10.1155/2019/9596421

**Published:** 2019-06-27

**Authors:** Harriët Wittink, Janke Oosterhaven, Jos Dekker, Cas Kruitwagen, Walter Devillé, Else Ellens, Carin Schroder

**Affiliations:** ^1^Research Group Lifestyle and Health, Utrecht University of Applied Sciences, Utrecht, Netherlands; ^2^Heliomare Rehabilitation Center, Wijk aan Zee, Netherlands; ^3^Julius Centre for Health Sciences and Primary Care, University Medical Center Utrecht, Utrecht, Netherlands; ^4^Amsterdam Institute of Social Science Research, University of Amsterdam, Amsterdam, Netherlands; ^5^De Hoogstraat Rehabilitation, Utrecht, Netherlands; ^6^Ecare4you, Amersfoort, Netherlands

## Abstract

**Background:**

The Treatment Beliefs Questionnaire has been developed to measure patients' beliefs of necessity of and concerns about rehabilitation. Preliminary evidence suggests that these beliefs may be associated with attendance of rehabilitation. The aim of this study was to translate and adapt the Treatment Beliefs Questionnaire for interdisciplinary pain rehabilitation and to examine the measurement properties of the Dutch translation including the predictive validity for dropout.

**Methods:**

The questionnaire was translated in 4 steps: forward translation from English into Dutch, achieving consensus, back translation into English, and pretesting on providers and patients. In order to establish structural validity, internal consistency, construct validity, and predictive validity of the questionnaire, 188 participants referred to a rehabilitation centre for outpatient interdisciplinary pain rehabilitation completed the questionnaire at the baseline. Dropout was measured as the number of patients starting, but not completing the programme. For reproducibility, 51 participants were recruited at another rehabilitation centre to complete the questionnaire at the baseline and one week later.

**Results:**

We confirmed the structural validity of the Treatment beliefs Questionnaire in the Dutch translation with three subscales, necessity, concerns, and perceived barriers. internal consistency was acceptable with ordinal alphas ranging from 0.66–0.87. Reproducibility was acceptable with ICC_2,1 agreement_ ranging from 0.67–0.81. Hypotheses testing confirmed construct validity, similar to the original questionnaire. Predictive validity showed the questionnaire was unable to predict dropouts.

**Conclusion:**

Cross-cultural translation was successfully completed, and the Dutch Treatment Beliefs Questionnaire demonstrates similar psychometric properties as the original English version.

## 1. Introduction

Interdisciplinary pain rehabilitation has been found to be effective at reducing medication use, reducing emotional distress, reducing health care utilization, reducing iatrogenic consequences, and increasing physical activity and return to work [1].

Despite its efficacy, pain rehabilitation nonadherence and dropout remain a major problem. A recent systematic review on interdisciplinary treatment of chronic musculoskeletal pain reports dropout ranging from 10 to 51% [2, 3] within the 8 included studies from the United States, Denmark, the Netherlands, and United Kingdom [4]. Dropout was defined as “patients with chronic pain, who were referred to a chronic pain management programme, who initiated (participated in the baseline assessments), but discontinued prior to completion of the entire programme” [5]. No high quality research was available on predictors of dropout and most predictors were only studied once.

According to the common sense model of self-regulation (CSM) [6–8] patients develop beliefs about their condition or illness which influence the interpretation of information and experiences and which guide behaviour [9]. Patients, therefore, bring preexisting beliefs about their illness and treatment (illness representations and treatment representations) to pain rehabilitation, which influence their evaluation of the treatment, their adherence, and even beneficial or adverse outcomes [10]. Several meta-analyses, however, have shown a very weak relationship between individual illness beliefs and adherence in patients with chronic diseases [11, 12]. Aujla et al. [12] report that an aspect of the CSM that has not been captured by their review, because of a lack of availability of data from included papers, concerns treatment beliefs. Beliefs about medications is one of the few treatment representations that have been studied systematically [8]. Research conducted with patients with a variety of long-term conditions suggests that the key beliefs influencing patients' common sense evaluations of prescribed medicines can be grouped under two categories: perceptions of personal need for treatment (necessity beliefs) and concerns about a range of potential adverse consequences [10, 13, 14]. This “Necessity-Concerns Framework (NCF)” potentially offers a convenient model for clinicians to elicit and address key beliefs underpinning patients' attitudes and decisions about treatment [10]. A recent meta-analytic review of the NCF about medicines prescribed for long-term conditions showed that higher adherence was associated with stronger perceptions of necessity of treatment and fewer concerns about treatment [10].

Compared to the body of evidence on treatment beliefs about medication, there is scant information on treatment beliefs about rehabilitation. Beliefs about rehabilitation have been shown to make a significant contribution to the prediction of rehabilitation outcomes in one study [15], and they are thought to strongly influence adherence to treatment [10, 14]. The Treatment Beliefs Questionnaire was initially developed as basis for predicting cardiac rehabilitation attendance after acute myocardial infarction. This 13 item questionnaire had good structural validity with internal consistencies >0.7 for all domains, resulting in acceptable construct validity [16]. An adapted version was used in elderly patients with COPD to test the association between treatment beliefs and baseline test performance and response to treatment [17]. For pain rehabilitation, no such questionnaire exists, even though treatment beliefs appear to be important in predicting adherence to treatment or dropout of patients with chronic pain entering an interdisciplinary pain rehabilitation programme. Several studies investigating adherence to cystic fibrosis, chronic obstructive pulmonary disease (COPD), and cardiac rehabilitation programmes have found that patients who expressed concerns about the programme or who reported practical barriers to attendance were less likely to attend [16–19].

Our first aim was to translate and adapt a questionnaire based on the NCF initially developed and validated for cardiac rehabilitation research [16, 20], later adapted and validated for use in an elderly COPD population [17] for patients attending interdisciplinary pain rehabilitation programmes. Our second aim was to describe the measurement properties of the translated and adapted treatment beliefs questionnaire, including the predictive validity for dropout.

## 2. Methods

### 2.1. Participant Recruitment

Consecutive patients with chronic noncancer pain who were referred to one of two rehabilitation centres (Heliomare, Wijk aan Zee and De Hoogstraat Revalidatie, Utrecht, the Netherlands) were invited to participate during the intake phase between October 2012 and October 2016. All participants provided informed consent and gave researchers permission to obtain sociodemographic and medical information from their medical records. Both rehabilitation centres conducted comparable interdisciplinary pain management programmes for patients with chronic pain, consisting of cognitive behavioural therapy with pain neuroeducation and exercise therapy. Patients who were judged appropriate candidates for the interdisciplinary programme by either a physiatrist (Heliomare) or a physiatrist, pain consultant, and a psychologist (Hoogstraat) were entered into the programme to start an initial period of assessment (diagnostic phase) by the other members of the team (psychologist, physical therapist, occupational therapist, social worker, sport professional, and music therapist) in order to come to an appropriate treatment plan (treatment phase). Excluded from the study were participants who were unable to read or write Dutch.

The study was registered with the Medical Ethics Committee of the Academic Medical Centre of Amsterdam which declared that it did not fall under the scope of the “Medical Research Involving Human Subjects Act” and by the internal research ethics review boards of the two rehabilitation centres. All patients provided written informed consent and were treated in accordance to the declaration of Helsinki [21].

### 2.2. Materials

The treatment beliefs questionnaire was developed by Cooper et al. [16] for patients referred to cardiac rehabilitation, based on the results from interview studies consistent with the NCF [20]. The questionnaire consists of 13 items across 4 domains: necessity (5 items), concerns (3 items), practical barriers (3 items), and perceived personal suitability (2 items). Internal consistency of the 4 domains varied between Cronbach's *α* = 0.70 for practical barriers and *α* = 0.79 for concerns. Items are scored on a 5-point Likert scale from 1 strongly disagree to 5 strongly agree. Higher scores on the necessity subscale indicate the patient is more likely to perceive treatment as necessary and to be clear as to how it will benefit. Higher scores on the concerns subscale indicate the patient has concerns about participating in treatment. Higher scores on practical barriers indicate there might be practical barriers to participating in treatment. A higher score on perceived personal suitability indicates a greater belief that (cardiac) rehabilitation is probably suitable for a younger, more active person.

In addition to the Treatment Beliefs Questionnaire, participants in the Heliomare programme completed the Dutch versions of the Brief Illness Perception Questionnaire (Brief IPQ) [22] and the Pain Self-Efficacy Questionnaire (PSEQ) [23]. The Brief IPQ has 8 dimensions (perceived consequences, timeline acute-chronic, amount of perceived personal control, treatment control, identity (symptoms) concern about the illness, coherence of the illness, and emotional representation) and uses one single item on a 0–10 scale to assess each dimension. The last item assesses causal perceptions by asking patients to list the three most likely causes for their illness. The PSEQ is a 10-item self-report questionnaire designed to assess the degree of confidence in performing a number of activities despite pain. Each item is rated on a 7-point Likert-type scale (0 = not at all confident, 6 = completely confident). Total scores range from 0 to 60 with a higher score indicating greater self-efficacy for functioning despite pain. Both Dutch versions of the Brief IPQ and the PSEQ have good psychometric properties [24, 25].

### 2.3. Procedure

For practical purposes, 51 participants were recruited between January and December 2014 in rehabilitation centre De Hoogstraat to complete the questionnaire at the baseline and one week later to determine reproducibility of the questionnaire. Participants completed the questionnaire before and after the one week diagnostic phase. Our assumption was that participants would remain stable during this period as treatment was not yet initiated. At the second administration, the participants and raters were not aware of the scores on the first administration. Test conditions were similar for all measurements.

For all other measurement properties (item-level analyses, structural validity, internal consistency, construct validity, and predictive validity), the participants completed the questionnaires at the baseline at the Heliomare Rehabilitation Centre.

### 2.4. Design and Analysis

For reproducibility, a test-retest was performed; for all other measurement properties, we used a prospective longitudinal design.

Data analysis was performed using SPSS version 23 (IBM Corp., Released 2012, IBM SPSS Statistics for Windows, Version 23.0. Armonk, NY), R statistical package version 3.1.1 [26], and Lisrel 8.8 (LISREL 8.80 for Windows, [27]). Questions 4 and 6 were reverse scored for all analyses. If the number of missings per domain was <2, missing item scores were replaced by the mean of the not missing items of the domain.

Means (SD) were calculated for the demographic data. Differences in age and gender between the two locations were tested by means of an independent *t*-test and chi-square analysis, respectively. Skewness tests were used to test for normal distribution on item level and domain level. To interpret skewness, we used the rule of thumb by Bulmer [28]. If skewness was less than −1 or greater than +1, the distribution was considered highly skewed. If skewness was between −1 and −½ or between +½ and +1, the distribution was moderately skewed. If skewness was between −½ and +½, the distribution was approximately symmetric.

In total, responses were missing on 9.4% of the items of the Treatment Beliefs Questionnaire. As all items had an abnormal distribution, we used polychoric correlations for item-level analyses. Per item no more than about 1% was missing.

#### 2.4.1. Item-Level Analyses

The distribution of item responses was determined by calculating the response option frequencies. Interitem correlations were determined using polychoric correlations, acknowledging that the 5-point Likert scale is in fact ordinal. The polychoric correlation coefficient is a measure of association for ordinal variables which rests upon an assumption of an underlying joint continuous distribution. It allows for other distributional assumptions than the joint normal distribution [29]. Correlations in the approximate range of 0.30–0.70 are desirable as lower values would indicate lack of homogeneity and high correlations would indicate item redundancy [30].

### 2.5. Structural Validity

Initially, an exploratory factor analysis (EFA) with promax rotation was applied to the correlation matrix of polychoric correlations to explore the dimensional structure of the Dutch pain Treatment Beliefs Questionnaire. Item loadings above 0.30 were used to retain items under one factor. Subsequently, confirmatory factor analyses (CFA) with a varimax rotation using the polychoric correlation matrix were performed to confirm the three domains of the Treatment Beliefs Questionnaire. We performed a confirmatory factor analysis using items 1–5 items for necessity (factor 1), items 6–9 for concerns (factor 2), and items 10 and 11 for practical barriers (factor 3) as reported in the literature [17], and we conducted a confirmatory factor analysis based on the results of our exploratory factor analysis using items 1–6 for necessity, items 7–9 for concerns, and items 10 and 11 for practical barriers. To determine how well the models fit to our data, we calculated the following: (1) the root mean square error of approximation (RMSEA): the RMSEA ranges from 0 to 1. A value of 0 indicates perfect fit. Hu and Bentler [31, 32] suggested ≤ 0.06 as a cutoff value for a good fit. (2) Comparative fit index (CFI): CFI values range from 0 to 1, with larger values indicating better fit. A CFI value ≥0.95 is accepted as an indicator of good fit [32]. (3) Goodness of fit index (GFI) and adjusted goodness of fit index (AGFI): the GFI and AGFI range between 0 and 1, with a value of >0.9 generally indicating acceptable model fit [33].

### 2.6. Internal Consistency

Internal consistency reliability measures the extent to which all items within a scale are indeed capturing the same construct. Ordinal alpha was calculated for the domains as established by the confirmatory factor analysis [34]. Alpha for a scale should not be smaller than 0.70 when used for research purposes, at least 0.80 for applied settings, and greater than 0.90 or even 0.95 for high-stake, individual-based educational, diagnostic, or clinical purposes [35].

#### 2.6.1. Reproducibility

As the data for the items were skewed, a quadratic weighted kappa was calculated as a measure of test-retest reliability for each item. Landis and Koch [36] proposed the following as standards for strength of agreement for the kappa coefficient: ≤0 = poor, 0.01–0.20 = slight, 0.21–0.40 = fair, 0.41–0.60 = moderate, 0.61–0.80 = substantial, and 0.81–1 = almost perfect. For test-retest reliability of the three domains (necessity, concerns, and practical barriers), we used a two-way random intraclass correlation (ICC_2,1 agreement_) as we considered sum scores of these domains to be at interval level. ICC values above 0.7 were considered to be acceptable [38]. To determine agreement, standard error of measurement ($\text{SEM}=\text{SD}\sqrt{\left(1-\text{ICC}\right)}$) was calculated using Cohen's formula for pooled SD [37]. The smallest detectable change for individuals was calculated $\left(\text{SDC}=1.96\;\times \;\sqrt{2}\times \text{SEM}\right)$, which reflects the smallest within-person change in score that, with *p* < 0.05, can be interpreted as a “real” change, above measurement error, in one individual (SDC_ind_) [38].

### 2.7. Construct Validity

Construct validity was tested by examining the correlations between the three subscales of the Treatment Beliefs Questionnaire and the Brief IPQ and the PSEQ. Based on previous research [16], we hypothesized there would be (1) medium positive correlations between the necessity domain and the IPQ item on treatment control, (2) medium positive correlations between concerns and IPQ consequences, IPQ concerns, and IPQ emotional response and a small negative correlation between Concerns and total PSEQ score, and (3) no or insignificant correlations between practical barriers and any of the IPQ items or the PSEQ.

We defined the strength of a correlation as anything smaller than 0.10 as insignificant; *r* = 0.10 to 0.29 small; *r* = 0.30 to 0.49 medium; and *r* = 0.50 to 1.0 large [39]. As the distribution of the IPQ item scores was skewed and the relationship with the necessity domain nonlinear, we used Spearman correlations. For the association between concerns and the PSEQ, we used a Pearson correlation (*r*_s_).

### 2.8. Predictive Validity

Finally, we tested the ability of the Treatment Beliefs Questionnaire to distinguish between dropouts and nondropouts. Dropout was defined as “patients with chronic pain, who were referred to a chronic pain management programme, who initiated (participated in the baseline assessments), but discontinued prior to completion of the entire programme” [5]. For this purpose, a receiver operating curve (ROC) and its area under the curve (AUC) was calculated for all three subscales.

## 3. Results

### 3.1. First Aim: Translation and Adaptation of the Treatment Belief Questionnaire

The Treatment Beliefs Questionnaire was translated in 4 stages by 2 translators (HW and CS) as recommended by Beaton et al. [40]. Both translators were bilingual and had expertise in the treatment of chronic pain. One translator, a psychologist (CS), was an expert in the common sense model of self-regulation. In stage 1, the two translators independently performed forward translations from English into Dutch; in stage 2, consensus by discussion was reached among the translators. In stage 3, the two translators independently translated the synthesized translation back into the original English language. In stage 4, we pretested the questionnaire on both health care providers and patients.

Two psychologists and 2 psychology assistants with expertise in treating patients with chronic pain, 2 pain consultants, and 2 experienced pain physical therapists were asked their opinion regarding the range and relevance of the questions. Their response to the range and relevance of items was positive, with one additional item suggested “in the days between the rehabilitation sessions, I am probably very tired from exercising” as proposed by Fischer et al. [17] who adapted the questionnaire for patients with COPD. For the perceived suitability questions, there was consensus that these questions were irrelevant as, among patients with chronic pain, age is not perceived to be a barrier to rehabilitation. One question of the practical barriers was dropped (“it would be financially difficult to take time off work to attend rehabilitation”) as it was felt this is not an issue in the Netherlands. The final questionnaire consisted of 11 items.

### 3.2. Pretesting of the Questionnaire

We pretested the 11 items using think-aloud techniques on 7 adults: 2 males and 5 females with a mean age of 40.7 years with chronic pain. Participants reported no difficulty comprehending the questions, but reported being surprised by the “very tired” question, as they were largely focused on pain. Participants also reported having difficulty completing the questionnaire as they did not know what to expect from pain rehabilitation, despite having had an educational group session on the content and goals of the pain rehabilitation programme.

Second aim: to describe the measurement properties of the translated treatment beliefs questionnaire, including the predictive validity for dropout.

A total of 208 consecutive patients were asked to participate in this study before the start of the clinical baseline assessment, of which 195 (94%) signed informed consent. Seven patients were excluded thereafter since they had no chronic musculoskeletal pain. Data on internal consistency and structural, construct, and predictive validity were collected at the baseline on 188 consecutive participants with chronic pain attending the chronic pain rehabilitation programme in the Heliomare Rehabilitation Centre. The sample was 70% female with a mean (SD) age of 47.0 (12) years. Mean (SD) pain intensity was 7.2 (1.5). Pain duration was between 0 and 5 years for 50.5% of the sample and more than 5 years for 38.3% of the sample. Data were missing on 11.2% of the sample. Thirty five participants (19%) dropped out during treatment.

In order to study reproducibility, 51 participants were included in rehabilitation centre “De Hoogstraat” who completed the treatment questionnaire twice. The sample had a mean (SD) age of 42.9 (11) years and was 67% female. There were no missing data for items 2, 3, 4, 8, and 9, one missing each for item 1 and items 6, 7, 10, and 11 and 4 missings both for items 4 and 5. Participants at De Hoogstraat Rehabilitation Centre were statistically significantly younger than participants at Heliomare Rehabilitation Centre (*p*=0.037). Chi-square testing showed no significant difference in gender between the sites (*p*=0.57). Chi-square testing also found no statistically significant differences in item distribution between the sites.

### 3.3. Item-Level Analyses

Descriptive analysis of the items demonstrated good distribution of response options (i.e., use of the entire scale) across all items, except the question “Attending pain rehabilitation may help me to do more activities” where no one scored “completely disagree.” No floor or ceiling effects were observed (see Table 1 for distributions).

Two of the 188 participants (1.1%) did not complete the Treatment Beliefs Questionnaire. Missing items were not included in the analysis. There were no missing items on the Brief IPQ or PSEQ.

The polychoric interitem correlations ranged between −0.01 and 0.76, indicating little item redundancy (see Table 2). Only one high interitem correlation (0.76) was observed between the two transportation items, but because these items inquire after different aspects of transportation (cost and availability), we decided to retain both items.

### 3.4. Structural Validity

The exploratory factor analysis (EFA) showed all factors loading above 0.3, with items 1–6 loading on one factor (necessity), items 7–9 loading on a second factor (concerns), and items 10 and 11 loading on a third factor (practical barriers). As Q6 (some aspects of the programme may be harmful to me) loaded on necessity, whereas this item should belong to the concerns domain according to the literature; we conducted two confirmatory factor analyses (CFA) to determine which model had a better fit.

CFA based on the literature with Items 1–5 loading on necessity, 6–9 on concerns, and items 10 and 11 on practical barriers showed a RMSEA = 0.077, CFI = 0.9, GFI 0.92, and AGFI = 0.87. The CFA based on the EFA with items 1–6 loading on necessity, 7–9 on concerns, and items 10 and 11 loading on practical barriers showed a RMSEA = 0.064, CFI = 0.94, GFI = 0.93, and AGFI = 0.89, indicating a slightly better fit to the data for the latter model (see Table 3).

### 3.5. Internal Consistency

Standardized ordinal alpha for practical barriers was 0.87. For necessity, ordinal alpha was 0.66, and for concerns, *α* = 0.66. We checked to see if alpha for the domains would increase if an item was dropped. This resulted in dropping the question about fatigue (item 9) from the concerns scale, which raised the overall alpha to 0.74.

The IPQ items and the treatment questionnaire domains were not distributed normally; therefore, we computed Spearman correlations to test our hypotheses.

We found small to medium associations between the three domains. High scores on necessity were related to low scores on concerns (*r*_s_ = −0.23), and we considered the association small. High scores on concerns were associated with high scores on practical barriers (*r*_s_ = 0.30). High scores on necessity domain were associated with low scores on practical barriers (*r*_s_ = −0.15).

### 3.6. Reproducibility

Reproducibility data for the three domains of the Treatment Beliefs Questionnaire are presented in Table 4.

Quadratic weighted kappa for the items ranged from fair; *κ* = 0.35 for “I have a clear picture of how pain rehabilitation will help me resume my daily activities” to substantial; *κ* = 0.72 for “I am worried that I may not be able to keep up with the exercise part.”

### 3.7. Construct Validity

The IPQ items and the treatment questionnaire domains were not distributed normally; therefore, we computed Spearman correlations to test our hypotheses.

Higher scores on the necessity domain were associated with higher scores on the Brief IPQ treatment control item (*r*_s_ = 0.39). Higher scores on the concerns domain were associated with higher scores on the Brief IPQ concerns item (*r*_s_ = 0.34). Associations between concerns and IPQ consequences (*r*_s_ = 0.25) and IPQ emotional response (*r*_s_ = 0.25) were considered small. Lower self-efficacy had a moderate association with higher scores on the concerns domain (*r*_s_ = −0.41). The associations (*r*_s_) between practical barriers and the IPQ items were all <0.10 and considered negligible. There was a small association between practical barriers and self-efficacy (PSEQ) *r*_s_ = −0.17.

### 3.8. Predictive Validity

Thirty-five (19%) patients dropped out at different phases of the treatment: 10 dropped out in the diagnostic phase and 25 dropped out in the treatment phase.

For nondropouts, the mean (SD) for necessity was 22.37 (3.0), concerns 4.9 (1.9), and practical barriers 3.59 (1.9). For dropouts, mean (SD) for necessity was 22.21 (3.0), concerns 5.03 (1.8), and practical barriers 4.26 (2.2). Mann–Whitney testing revealed no statistically significant differences between nondropouts and dropouts.

To determine the predictive validity for dropout (yes/no) of the treatment beliefs questionnaire, we calculated a ROC curve and the area under the curve (AUC) for each domain.

**Figure 1:**
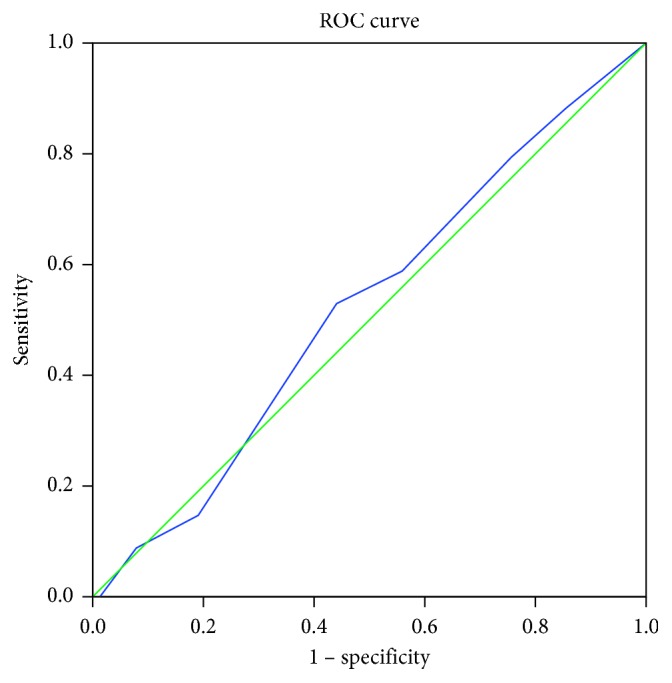
ROC curve of the domain concerns. Diagonal segments are produced by ties.

**Figure 2:**
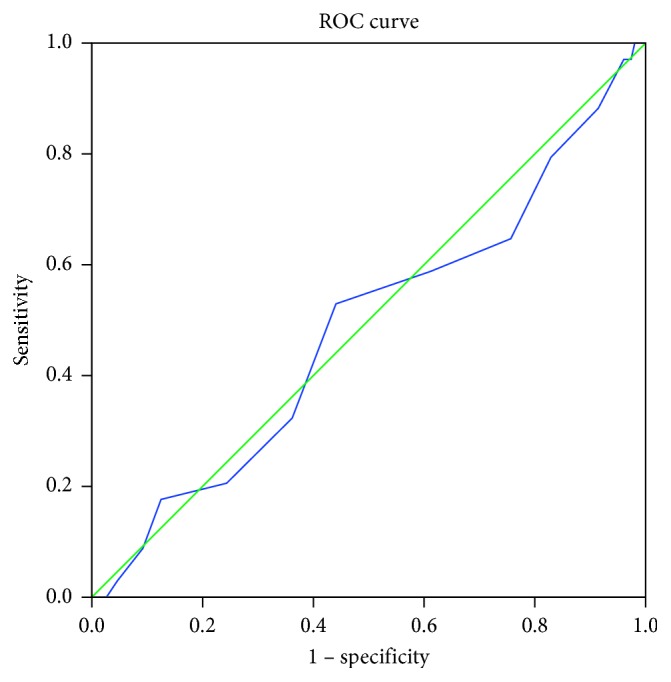
ROC curve of the domain necessity. Diagonal segments are produced by ties.

**Figure 3:**
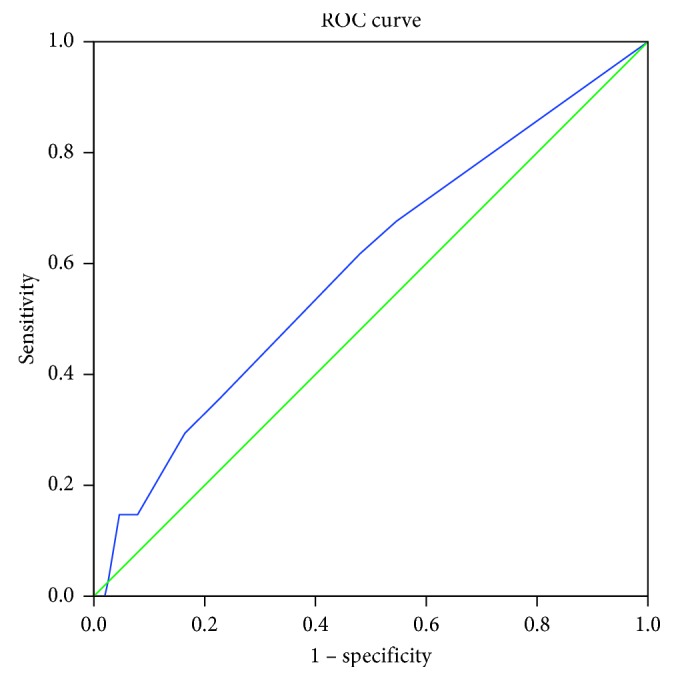
ROC cure of the domain practical barriers. Diagonal segments are produced by ties.

The AUC for necessity was 0.515 (95% CI 0.40–0.63) with a standard error (SE) of 0.057. For concerns, AUC (SE) was 0.522 (0.053), 95% CI 0.42–0.63, and for practical barriers, AUC (SE) was 0.592 (0.055), 95% CI 0.48–0.70. As the AUCs were poor and showed no predictive validity, we did not calculate sensitivity and specificity (Figures 1–3).

## 4. Discussion

The first aim of the study was to translate and adapt the Treatment Beliefs Questionnaire as developed by Cooper et al. [16] for Dutch patients with chronic pain attending interdisciplinary pain rehabilitation. We did so in a 4 step process, which ultimately resulted in an 11 item questionnaire.

The perceived suitability questions from the original questionnaire were dropped as there was consensus that these questions were irrelevant to pain rehabilitation. One question of the practical barriers domain was dropped (“it would be financially difficult to take time off work to attend rehabilitation”) as it was felt this is not an issue in the Netherlands.

In the think-aloud study, patients indicated being surprised by the “very tired” item, as they were largely focused on pain. We left the item in, as it was deemed to be important by their providers. However, in the statistical analysis, we had to drop the item, as it lowered the alpha on the concerns subscale. Participants indicated difficulty completing the questionnaire as they did not quite know what to expect from the pain programme, despite them having had an educational session of 1 hour on the content and purpose of the chronic pain rehabilitation programme. This was evidenced by the high number of “neutral” answers on, for instance, the “Some aspects of the pain rehabilitation programme are unnecessary for me” item. An exception was item 3: “Attending pain rehabilitation may help me to do more activities,” where 86% of patients scored agree or completely agree, which may be a reflection of the desired outcome of the chronic pain programme by patients.

The second aim of this study was to determine the measurement properties of the Treatment Beliefs questionnaire. Structural validity testing revealed three subscales (domains) representing necessity, concerns, and practical barriers. In contrast to the original work by Cooper et al. [16], we found that item 6 “some aspects of the pain programme may be harmful to me” loaded better on the necessity subscale than on the concerns subscale. This may be due to the fact that about 96% of respondents scored disagree, disagree completely, or neutral, indicating no particular concerns about the potential harmfulness of the pain programme. This was surprising given the body of knowledge on fear of movement in patients with chronic pain [41].

Internal consistency was fair to good with alphas ranging from 0.66–0.87. This is comparable to the findings by Fischer et al. [17] and Cooper et al. [16]. Considering the low interitem correlations of the necessity subscale, it is not surprising that the internal consistency was only fair. This may be an indication of dissimilar beliefs (on return to work, do more activities, and necessity of parts of the pain programme) contributing to the necessity subscale.

Reproducibility was acceptable with a small measurement error for both the necessity and concerns subscales. Reproducibility of the practical barriers subscale was fair, probably due to the lack of heterogeneity of answers; about 75% of respondents answered disagree or completely disagree.

In testing our hypotheses for construct validity, we found similar size and consistent correlations, in the same direction as Cooper et al. [16], confirming construct validity. Patients who perceived the pain programme as necessary had stronger beliefs in the treatment. Patients with higher concerns about the pain programme were more concerned about their condition, perceived their condition would last a long time, and were more affected emotionally. Patients with lower pain self-efficacy had higher perceived concerns about treatment and higher perceived practical barriers.

ROC analysis showed no predictive validity for dropout with AUC < 0.6. Fischer et al. [17] also found no difference in participants' treatment beliefs between dropouts and participants who completed the programme. Cooper et al. [16] on the other hand found a significant difference in the necessity beliefs subscale between those intending and not intending to participate in the (cardiac) rehabilitation programme before hospital discharge. It is possible that the Treatment Beliefs Questionnaire is able to distinguish between those who are referred to the pain programme and attend and those who are referred, but do not attend. The Treatment Beliefs Questionnaire might help health professionals to identify patients who are likely not to attend the programme and who might need extra explanation before they are entered into the programme.

This is the first study on treatment beliefs, using the NCF, in a sample of Dutch participants attending pain rehabilitation. It has been argued that the NCF might work well for medication use [14], while for other treatments, perceived credibility and treatment expectancy have been considered more relevant [42, 43]. Treatment credibility has been associated with outcomes in a combined physical and cognitive behavioural treatment in chronic low back pain [44], with dropout in an Internet-based cognitive behavioural relaxation programme [45] and a face-to-face and internet-based cognitive behavioural therapy for bulimia nervosa [46]. Comparing the constructs of the NCF with treatment credibility and expectancy could be subject for further study.

There are several limitations to this study. The same two translators conducted the forward and backwards translation. This may be a source of bias. Another limitation of this study is the location and time span for reproducibility testing. For practical purposes, we conducted the reproducibility study at the Hoogstraat Rehabilitation Centre, while all other data were collected in the Heliomare Rehabilitation Centre. Although the programmes are similar, there were some differences between participants, as the participants in the Hoogstraat Rehabilitation Centre were somewhat younger. A time interval of about 2 weeks is often considered appropriate for the evaluation of reproducibility of a patient reported outcome instrument if the patients are stable [30]. To ensure that our participants remained stable, we tested before and after the one week where patients underwent further evaluation before treatment began. Although participants had no insight into their earlier responses, recall bias cannot be excluded.

## 5. Conclusion

We confirmed the structural validity of the Dutch translation of the Treatment beliefs Questionnaire for chronic pain rehabilitation with three subscales: necessity, concerns, and perceived barriers. Internal consistency was acceptable, as was reproducibility. Hypotheses testing confirmed construct validity and predictive validity showed the questionnaire was unable to predict dropouts. Cross-cultural translation was successfully completed, and the Dutch Treatment Beliefs Questionnaire demonstrates similar psychometric properties as the original English version. This questionnaire may be a clinically useful tool to identify patients' concerns about and possible barriers for chronic pain rehabilitation. We recommend these are discussed in the diagnostic phase of treatment to eliminate any possible concerns about and barriers for pain rehabilitation.

## Figures and Tables

**Table 1:** Item response option distributions in %.

Question	Completely disagree	Disagree	Neutral	Agree	Completely agree	Missing
1. I have a clear picture of how pain rehabilitation will help me resume my daily activities	**3.2**	**4.8**	**46.8**	**34**	**10.6**	**0.5**
	0	3.9	29.4	58.8	5.9	2
2. I have a clear picture of what I want to achieve by attending pain rehabilitation	**1.1**	**2.7**	**30.9**	**51.1**	**13.8**	**0.5**
	0	2.0	17.6	56.9	23.5	0
3. Attending pain rehabilitation may help me to do more activities	**0**	**2.1**	**11.2**	**50.5**	**35.6**	**0.5**
	0	2.0	13.7	54.9	29.4	0
4. Some aspects of pain rehabilitation are unnecessary for me	**16**	**16.5**	**62.8**	**3.2**	**1.1**	**0.5**
	2.0	2.0	51.0	25.5	11.8	7.8
5. I hope that attending pain rehabilitation may help me to return to (volunteer) work quickly	**5.3**	**1.6**	**38.8**	**30.3**	**23.4**	**0.5**
	7.8	5.9	31.4	27.5	19.6	7.8
6. Some aspects of pain rehabilitation may be harmful to me	**31.9**	**25.5**	**37.2**	**3.7**	**0.5**	**1.1**
	31.4	47.1	15.7	2.0	2.0	2
7. I am worried that I may not be able to keep up with the exercise part	**23.4**	**20.7**	**34**	**19.1**	**1.6**	**1.1**
	23.5	39.2	19.6	13.7	2.0	2.0
8. I may not be physically fit enough to attend pain rehabilitation	**20.2**	**33.5**	**33.5**	**10.6**	**1.1**	**1.1**
	29.4	41.2	21.6	7.8	0	0
9. On the days between the rehabilitation sessions, I am probably very tired from exercising	**7.4**	**12.2**	**42.6**	**25.5**	**11.2**	**1.1**
	7.8	21.6	39.2	25.5	5.9	0
10. The cost of transport may prevent me from attending pain rehabilitation	**46.8**	**28.7**	**15.4**	**3.7**	**4.3**	**1.1**
	58.8	33.3	2.0	2.0	2.0	2
11. Availability of transport will influence my decision to attend pain rehabilitation	**51.1**	**26.1**	**12.2**	**7.4**	**2.1**	**1.1**
	58.8	33.3	3.9	2.0	0	0

*Note*. In bold, distribution from Heliomare (*n*=188), underneath the distribution from the Hoogstraat (*n*=51).

**Table 2:** Polychoric interitem correlations (*n*=188).

Question	Q1	Q2	Q3	Q4	Q5	Q6	Q7	Q8	Q9	Q10	Q11
1. I have a clear picture of how pain rehabilitation will help me resume my daily activities	1.0	0.41	0.22	−0.17	0.11	−0.24	−0.19	−0.10	0.02	−0.02	0.01
2. I have a clear picture of what I want to achieve by attending pain rehabilitation	0.41	1.0	0.26	−0.26	0.28	−0.23	−0.09	−0.02	0.09	−0.12	−0.16
3. Attending pain rehabilitation may help me to do more activities	0.22	0.26	1.0	−0.13	0.41	−0.29	−0.12	−0.12	−0.03	–0.20	−0.15
4. Some aspects of pain rehabilitation are unnecessary for me^*∗*^	−0.17	−0.26	−0.13	1.0	−0.15	0.33	0.20	0.17	0.11	0.10	0.17
5. I hope that attending pain rehabilitation may help me to return to work quickly	0.11	0.28	0.41	−0.15	1.0	−0.20	−0.14	−0.13	0.04	−0.06	−0.09
6. Some aspects of pain rehabilitation may be harmful to me	−0.24	−0.23	−0.29	0.33	−0.20	1.0	0.30	0.24	0.03	0.27	0.27
7. I am worried that I may not be able to keep up with the exercise part	−0.19	−0.09	−0.12	0.20	−0.14	0.30	1.0	0.59	0.34	0.29	0.31
8. I may not be physically fit enough to attend pain rehabilitation	−0.10	−0.02	−0.12	0.17	−0.13	0.24	0.59	1.0	0.25	0.32	0.33
9. On the days between the rehabilitation sessions, I am probably very tired from exercising	−0.02	−0.09	−0.03	0.11	0.04	0.03	0.34	0.25	1.0	0.12	0.16
10. The cost of transport may prevent me from attending pain rehabilitation	−0.02	−0.12	−0.20	0.10	−0.06	0.27	0.29	0.32	0.12	1.0	0.76
11. Availability of transport will influence my decision to attend pain rehabilitation	0.01	−0.16	−0.15	0.17	−0.09	0.27	0.31	0.33	0.16	0.76	1.0

**Table 3:** Results of confirmatory factor analysis based on exploratory factor analysis.

Loadings	Factor 1 (necessity)	Factor 2 (concerns)	Factor 3 (practical barriers)
1. I have a clear picture of how pain rehabilitation will help me resume my daily activities	0.639	0	0
2. I have a clear picture of what I want to achieve by attending pain rehabilitation	0.771	0	0
3. Attending pain rehabilitation may help me to do more activities	0.683	0	0
4. Some aspects of pain rehabilitation are unnecessary for me	−0.587	0	0
5. I hope that attending pain rehabilitation may help me to return to (volunteer) work quickly	0.567	0	0
6. Some aspects of pain rehabilitation may be harmful to me	−0.657	0	0
7. I am worried that I may not be able to keep up with the exercise part	0	0.926	0
8. I may not be physically fit enough to attend pain rehabilitation	0	0.733	0
9. On the days between the rehabilitation sessions, I am probably very tired from exercising	0	0.450	0
10. The cost of transport may prevent me from attending pain rehabilitation	0	0	0.861
11. Availability of transport will influence my decision to attend pain rehabilitation	0	0	0.954

**Table 4:** Reproducibility Treatment Beliefs Questionnaire.

Domains	T1 (mean, SD)	T2 (mean, SD)	ICC_2,1_ 95% CI	SEM	SDC_ind_
Necessity	22.69 (2.54)	23.28 (2.72)	0.687 0.50–0.81	1.77	4.92
Concerns	4.40 (1.77)	4.22 (1.62)	0.81 0.69–0.89	0.91	2.55
Practical barriers	3.0 (1.41)	2.86 (1.25)	0.665 0.48–0.79	0.96	2.67

*Note*. Replacing missing data by the mean score of the domains yielded the same results. ICC_2,1_: two-way random effects intraclass correlation coefficient; SEM: standard error of measurement; SDC_ind_: smallest detectable change for an individual.

## Data Availability

The data used to support the findings of this study are available from the corresponding author upon reasonable request.
